# Tissue-specific distribution of hemicelluloses in six different sugarcane hybrids as related to cell wall recalcitrance

**DOI:** 10.1186/s13068-016-0513-2

**Published:** 2016-05-04

**Authors:** Thales H. F. Costa, Miguel E. Vega-Sánchez, Adriane M. F. Milagres, Henrik V. Scheller, André Ferraz

**Affiliations:** Departamento de Biotecnologia, Escola de Engenharia de Lorena, Universidade de São Paulo, Lorena, SP 12602-810 Brazil; The Joint BioEnergy Institute, Lawrence Berkeley National Laboratory, 1 Cyclotron Rd., Berkeley, CA 94720 USA; Monsanto Company, 800N. Lindbergh Blvd., Creve Coeur (St. Louis), MO 63141 USA

**Keywords:** Recalcitrance, Mixed-linkage glucans, Immunofluorescence, Sugarcane

## Abstract

**Background:**

Grasses are lignocellulosic materials useful to supply the billion-tons annual requirement for renewable resources that aim to produce transportation fuels and a variety of chemicals. However, the polysaccharides contained in grass cell walls are built in a recalcitrant composite. Deconstruction of these cell walls is still a challenge for the energy-efficient and economically viable transformation of lignocellulosic materials. The varied tissue-specific distribution of cell wall components adds complexity to the origins of cell wall recalcitrance in grasses. This complexity usually led to empirically developed pretreatment processes to overcome recalcitrance. A further complication is that efficient pretreatment procedures generally treat the less recalcitrant tissues more than necessary, which results in the generation of undesirable biomass degradation products.

**Results:**

Six different sugarcane hybrids were used as model grasses to evaluate the tissue-specific distribution of hemicelluloses and the role of these components in cell wall recalcitrance. Acetylated glucuronoarabinoxylan (GAX) occurs in all tissues. Mixed-linkage glucan (MLG) was relevant in the innermost regions of the sugarcane internodes (up to 15.4 % w/w), especially in the low-lignin content hybrids. Immunofluorescence microscopy showed that xylans predominated in vascular bundles, whereas MLG occurred mostly in the parenchyma cell walls from the pith region of the hybrids with low-lignin content. Evaluation of the digestibility of sugarcane polysaccharides by commercial enzymes indicated that the cell wall recalcitrance varied considerably along the internode regions and in the sugarcane hybrids. Pith regions of the hybrids with high MLG and low-lignin contents reached up to 85 % cellulose conversion after 72 h of hydrolysis, without any pretreatment.

**Conclusions:**

The collective characteristics of the internode regions were related to the varied recalcitrance found in the samples. Components such as lignin and GAX were critical for the increased recalcitrance, but low cellulose crystallinity index, high MLG contents, and highly substituted GAX contributed to the generation of a less recalcitrant material.

**Electronic supplementary material:**

The online version of this article (doi:10.1186/s13068-016-0513-2) contains supplementary material, which is available to authorized users.

## Background

The recalcitrance of lignocellulosic materials limits the enzymatic conversion of cell wall polysaccharides into fermentable sugars. Many aspects of plant cell walls, such as the embedding of cellulose microfibrils by hemicelluloses and lignin, contribute to hindering the enzymes’ access to the cellulose chains [1–3]. Crystalline cellulose by itself limits the action of the enzymes to the external surfaces of the crystal [4], making cellulose amorphogenesis necessary before complete deconstruction of the polymer by enzymes [5, 6].

The tissue-specific distribution of lignin and hemicelluloses adds complexity to the origins of cell wall recalcitrance. Monocot grasses are particularly useful models to evaluate tissue-specific recalcitrance because their cell diversity is greater than in the xylem of woody dicots. For example, sugarcane and maize internodes have been evaluated according to the tissue-specific distribution of lignin indicating that highly lignified vessel and fiber cell walls from the vascular bundles are significantly more recalcitrant than the less lignified parenchyma [7–11].

In the case of hemicelluloses, the amount of polysaccharides and their decoration pattern have been associated with varied recalcitrance in a wide range of materials and tissues. Hemicellulose removal from the cell walls by chemical or enzymatic pretreatments has been proven to increase cell wall digestibility [2, 12–14]. The decoration pattern was also demonstrated to affect the primary hydrolysis of hemicelluloses by enzymes. In general, highly branched polymers are more resistant than less decorated polymers [2, 15]. This behavior contrasts with cell wall recalcitrance derived from cellulose–hemicellulose interactions. For example, the most abundant hemicellulose in grasses is acetylated glucuronoarabinoxylan (GAX), a polysaccharide with a xylan backbone containing acetyl, glucuronyl, and arabinosyl side groups [3]. The polysaccharide occurs in primary and secondary cell walls, although it differs in the substitution pattern. GAX from secondary cell walls presents fewer side groups than those from primary cell walls, which allows for stronger interactions between secondary wall GAX and cellulose [16]. This strong cellulose–hemicellulose interaction perhaps contributes to the increased recalcitrance observed in secondary cell walls.

Mixed-linkage glucan (MLG), a non-branched polymer of glucose residues with ß-1, 4 and ß-1, 3 linkages, occurs in grasses and related Poales families [16, 17]. Due to its amorphous characteristic, MLG has been a target in the cell wall engineering of easily digestible biomasses [18]. MLG is usually correlated with cell wall expansion in grasses, although its role in cell walls is not fully understood [19]. This polysaccharide usually accumulates in primary cell walls from elongation zones and meristem, as described in grass coleoptiles and roots [20, 21]. However, MLG has also been immunodetected in the secondary cell walls of mature tissues of grasses [22, 23].

The manner in which the factors discussed above contribute to recalcitrance in the lignified cell walls still requires attention. Past and recent experimental efforts have shown that cell maturation stage and the diverse distribution of cell wall components along the grass stems provide good models to study recalcitrance in grasses [7–10, 24–26]. In sugarcane, we have shown that the center of sugarcane stems can be rapidly hydrolyzed by commercial enzymes without pretreatment. On the other hand, the outermost regions of the stem are very recalcitrant, requiring lignin and/or hemicellulose removal prior to efficient enzymatic hydrolysis of the polysaccharides [8, 27].

After evaluating a number of sugarcane hybrids, we suggested that the fibers and vessels contained in the vascular bundles are recalcitrant due to low cellulose availability, measured as the glucan content divided by the sum of lignin plus hemicellulose. In contrast, less lignified parenchyma cell walls present in the pith region were less recalcitrant because they presented enhanced glucan content and fewer cellulose-embedding components [26]. In the present work, the type of hemicellulose and their tissue-specific distribution were evaluated in six different sugarcane hybrids. The internodes of each sugarcane plant were further divided into three different fractions from pith to the outermost region. Hemicellulose composition, immunomapping using fluorescence microscopy, and the cell wall crystallinity index were determined together with the cell wall digestibility by commercial enzymes. The compiled data demonstrated that the high recalcitrance of vascular bundles found mostly in the outermost regions of the stem can be assigned to several cell wall characteristics, such as high contents of lignin and xylan, low MLG contents, the presence of less substituted xylan structures, and a high proportion of crystalline cellulose. In contrast, tissues found in the pith region were less recalcitrant because they were poorer in lignin, richer in MLG, presented lower proportion of crystalline cellulose and more substituted xylans.

## Results and discussion

### Chemical composition of the sugarcane internodes

Six sugarcane hybrids that contain varied lignin and hemicellulose contents were dissected into three internode fractions: pith, pith-rind interface, and rind. Lignocellulosic biomass from these samples was characterized according to chemical composition as shown in Table 1. Pith regions were rich in glucan (40–55 %) and poor in lignin (14–22 %). Rind fractions, on the other hand, showed higher contents of lignin (20–22 %) and proportionally less glucan (40–44 %). Xylan content was the lowest in pith fractions (14–20 %), increasing in content toward the rind region (20–23 %).

**Table 1:** Chemical composition of internode fractions from six different sugarcane hybrids

Sugarcane hybrids	Internode region	Chemical composition (%, w/w on oven dry basis)							
		Ethanol-soluble extractives	Glucan	Xylan	Arabinosyl	Acetyl	Lignin		
							Acid insoluble	Acid soluble	Total lignin
H 89	Pith	1.4 ± 0.2	55.0 ± 0.1	14.5 ± 0.1	3.7 ± 0.1	2.7 ± 0.1	11.0 ± 0.5	2.9 ± 0.3	13.9 ± 0.4
	Interface	2.2 ± 0.1	46.5 ± 0.3	18.9 ± 0.2	2.0 ± 0.1	3.5 ± 0.1	17.3 ± 0.2	3.2 ± 0.3	20.5 ± 0.3
	Rind	2.6 ± 0.1	43.7 ± 0.3	22.7 ± 0.1	1.4 ± 0.1	3.7 ± 0.1	19.4 ± 0.5	2.3 ± 0.2	21.7 ± 0.4
H 146	Pith	3.2 ± 0.1	44.1 ± 0.5	20.4 ± 0.1	2.6 ± 0.1	4.1 ± 0.1	15.0 ± 0.4	2.9 ± 0.1	17.9 ± 0.3
	Interface	4.8 ± 0.2	38.1 ± 0.9	20.7 ± 0.6	1.8 ± 0.1	4.2 ± 0.2	18.8 ± 0.1	3.3 ± 0.4	22.0 ± 0.3
	Rind	4.3 ± 0.4	43.6 ± 0.1	21.3 ± 0.1	1.1 ± 0.1	3.6 ± 0.1	18.9 ± 0.2	2.8 ± 0.4	21.6 ± 0.3
H 58	Pith	1.4 ± 0.2	51.8 ± 0.1	17.5 ± 0.1	2.9 ± 0.1	3.9 ± 0.3	10.2 ± 0.2	4.1 ± 0.7	14.3 ± 0.5
	Interface	3.4 ± 0.2	47.0 ± 0.1	19.9 ± 0.1	2.4 ± 0.1	4.0 ± 0.1	14.3 ± 0.9	2.7 ± 0.2	17.0 ± 0.6
	Rind	3.0 ± 0.2	44.1 ± 0.2	22.5 ± 0.2	1.4 ± 0.1	4.3 ± 0.3	16.8 ± 0.7	3.3 ± 0.1	20.1 ± 0.5
H 166	Pith	3.3 ± 0.2	50.0 ± 0.1	17.6 ± 0.1	2.1 ± 0.1	3.4 ± 0.1	14.1 ± 0.1	2.7 ± 0.1	16.9 ± 0.1
	Interface	5.1 ± 0.1	42.1 ± 0.6	20.1 ± 0.2	1.6 ± 0.1	3.6 ± 0.4	18.0 ± 0.1	2.8 ± 0.1	20.8 ± 0.1
	Rind	3.2 ± 0.1	43.8 ± 0.1	20.4 ± 0.3	1.2 ± 0.1	3.7 ± 0.1	19.2 ± 0.1	2.4 ± 0.1	21.6 ± 0.1
H 321	Pith	5.1 ± 0.1	43.1 ± 0.1	19.6 ± 0.5	2.9 ± 0.2	3.7 ± 0.1	16.9 ± 0.1	3.2 ± 0.1	20.1 ± 0.1
	Interface	2.4 ± 0.2	38.5 ± 0.2	20.2 ± 0.1	1.9 ± 0.1	4.1 ± 0.1	20.8 ± 0.1	3.0 ± 0.2	23.8 ± 0.1
	Rind	4.0 ± 0.1	42 ± 2	20.7 ± 0.7	1.4 ± 0.1	3.8 ± 0.1	19.5 ± 0.2	2.4 ± 0.1	22.0 ± 0.2
H 140	Pith	3.6 ± 0.1	39.9 ± 0.4	19.2 ± 0.2	2.0 ± 0.1	3.0 ± 0.3	19.0 ± 0.2	2.8 ± 0.5	21.9 ± 0.4
	Interface	2.5 ± 0.3	40.8 ± 0.1	21.6 ± 0.1	1.8 ± 0.1	4.6 ± 0.1	17.5 ± 0.9	3.2 ± 0.8	20.7 ± 0.8
	Rind	3.1 ± 0.1	40.5 ± 0.7	22.1 ± 0.4	1.3 ± 0.1	3.1 ± 0.4	19.8 ± 0.2	2.6 ± 0.1	22.4 ± 0.2

The detailed occurrence of hemicelluloses in the studied hybrids was initially assessed through a mild TFA hydrolysis procedure, which barely hydrolyzes crystalline cellulose and therefore allows for the evaluation of non-cellulosic monosaccharides [28]. The results showed that xylose (9–19 %), followed by glucose (1–12 %) and arabinose (1.7–3.9 %), was the most abundant monosaccharide released by mild TFA hydrolysis (Additional file 1: Table S1). Lower amounts of xylose released in the TFA hydrolysates (Additional file 1: Table S1) compared to the sulfuric acid hydrolysates (Table 1) suggest that hemicellulose hydrolysis by TFA was incomplete under the evaluated reaction conditions. Several other monosaccharides were also detected in minor amounts in TFA hydrolysates, including galactose (0.2–1.1 %), rhamnose (0.07–0.16 %), galacturonic acid (0.2–0.6 %), and trace of glucuronic acid. Peaks corresponding to 4-O-methylglucuronic acid were also detected but not quantified. The detection of galactose, rhamnose, and galacturonic acid indicated the presence of pectin in the samples [29], although in small amounts within the cell walls of the sugarcane samples. TFA hydrolysis of the samples corroborated with Table 1 data indicating that acetyl-substituted GAX predominates in the hemicelluloses from sugarcane with xylan contents increasing from pith to rind internode regions, independent of the studied hybrid. Interestingly, the arabinose levels decreased from pith to rind, indicating that rind-GAXs are less decorated with arabinosyl groups than pith-GAXs.

**Fig. 1:**
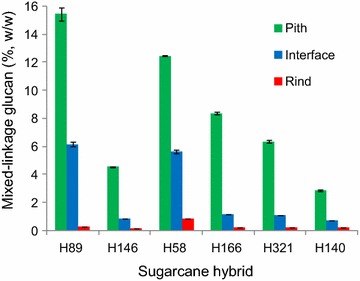
MLG contents in pith, pith-rind interface, and rind regions of six different sugarcane hybrids. *Error bars* represent the standard deviations for triplicate determinations

A remarkable result from TFA hydrolysates was that the pith samples had high glucose contents. This result suggested the presence of a glucose-rich labile polysaccharide in the hemicellulosic fraction of some sugarcane hybrids, especially in the pith region. Xyloglucan and glucomannan can usually be found in the primary cell walls of grasses, but in low amounts [30]. In contrast, MLG occurs in grasses in larger amounts [17, 23]. Immunoassays and enzyme-based quantification revealed that MLG was indeed abundant in the sugarcane samples, with differential distribution along the internode regions (Additional file 2: Figure S1; Fig. 1, respectively). Pith regions showed the highest MLG amounts (3–15 %), especially in sugarcane hybrids with low-lignin contents, such as H89 and H58 (Fig. 1). The MLG content decreased in the pith-rind interface fractions (1–6 %) and was a minor component in the rind (0.1–1 %), in agreement with the glucose distribution observed in the TFA hydrolysates (Additional file 1: Table S1). This differential distribution of MLG was confirmed by dot-blotting immunoassays using a monoclonal antibody recognizing (1–3, 1–4)-ß-d-glucan epitopes (Additional file 2: Figure S1). The results showed strong labeling for KOH extracts recovered from pith and the pith-rind interface and scarce or no labeling for rind extracts.

MLG predominance in the pith seems to be related to a great number of parenchyma cells found in this region of sugarcane internodes [8, 26]. As MLG has been found mostly in primary cell walls of grasses, especially in elongating and parenchyma tissues [20–22], a large number of parenchyma cells could explain the higher concentration of this polysaccharide in the center of sugarcane internodes. On the other hand, the rind showed no or very low MLG contents, which seems related to the presence of highly lignified tissues. MLG is known to be growth stage dependent in coleoptiles because it decreases as elongation ceases [31–34]. MLG is largely degraded and replaced by GAX in mature tissues and secondary cell walls of grasses [30]. Taken together, these observations suggest that some of the studied sugarcane hybrids had inner tissues still in an incomplete maturation phase despite being 12-month old.

### Xylan and MLG immunolocalization

**Fig. 2:**
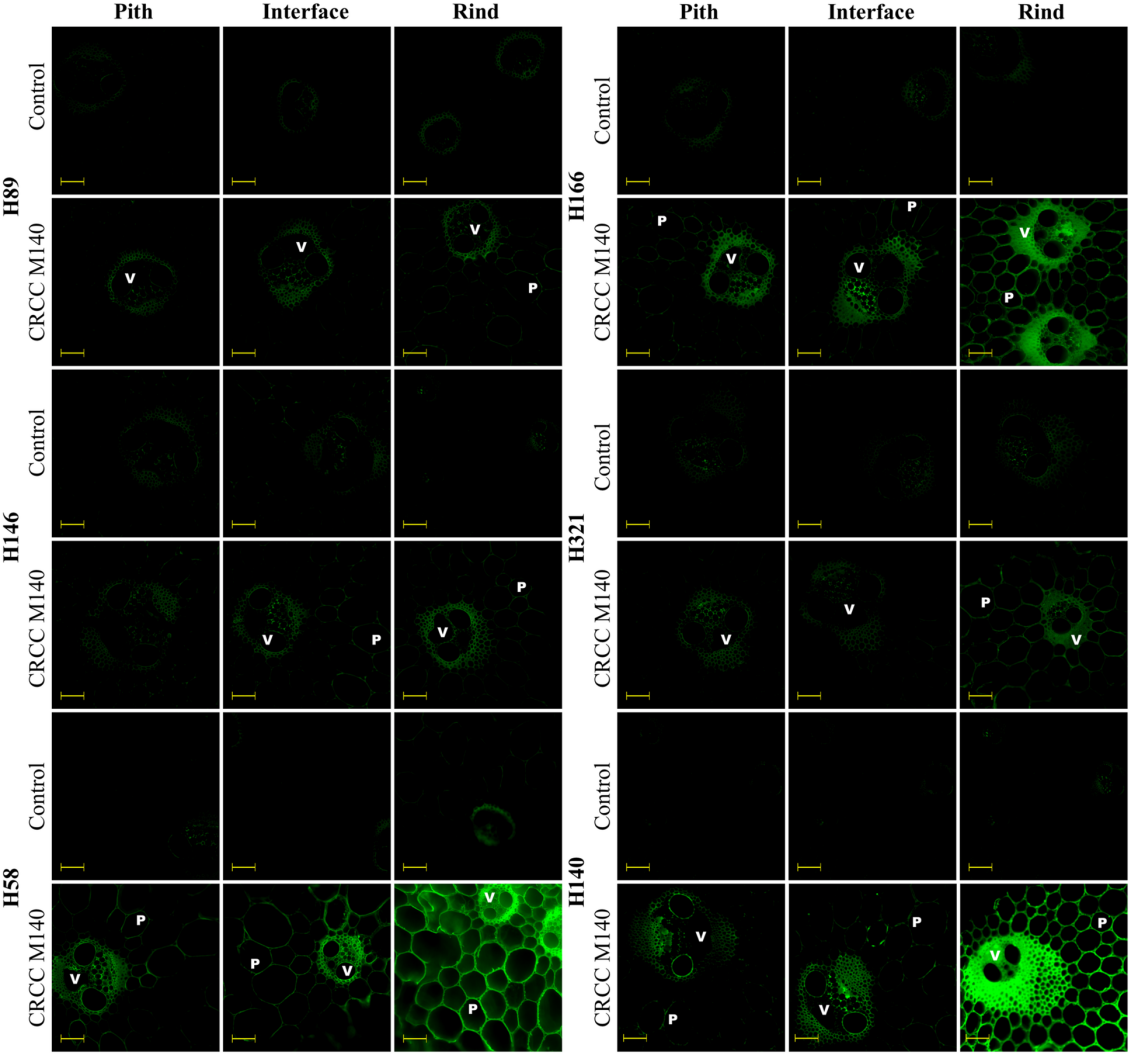
Fluorescence micrographs of pith, pith-rind interface, and rind transversal cuts of six different sugarcane hybrids based on indirect immunolabeling analysis for xylan epitopes labeled with CRCC M140 primary antibody and Alexa Fluor 514 secondary antibody. *V* and *P* indicate vascular bundles and parenchyma, respectively. Control corresponds to the transversal cuts labeled only with the secondary antibody. *Scale bars* correspond to 100 µm

The sugarcane hybrids were investigated by immunofluorescence techniques to determine xylan and MLG distribution among different tissues and cell types. Cross sections of each sample were treated with primary monoclonal antibodies able to detect xylan epitopes or (1–3, 1–4)-ß-d-glucan epitopes, followed by a secondary antibody containing the fluorochrome component. Xylan epitopes (based on CRCC-M140 antibody) [35] were differentially distributed along the internode regions and tissues of the studied sugarcane samples (Fig. 2). The fluorescence intensity increased from pith to rind in most hybrids. Labeling was notably stronger in vascular bundles, especially fiber cell walls from the rind, indicating a great deposition of xylan in this tissue. Parenchyma cell walls were barely labeled by CRCC-M140 antibody in pith but appeared labeled in rind. This distribution of xylan over different internode regions is in accordance with the xylose distribution detected in the sulfuric acid and TFA hydrolysates (Table 1; Additional file 1: Table S1, respectively).

**Fig. 3:**
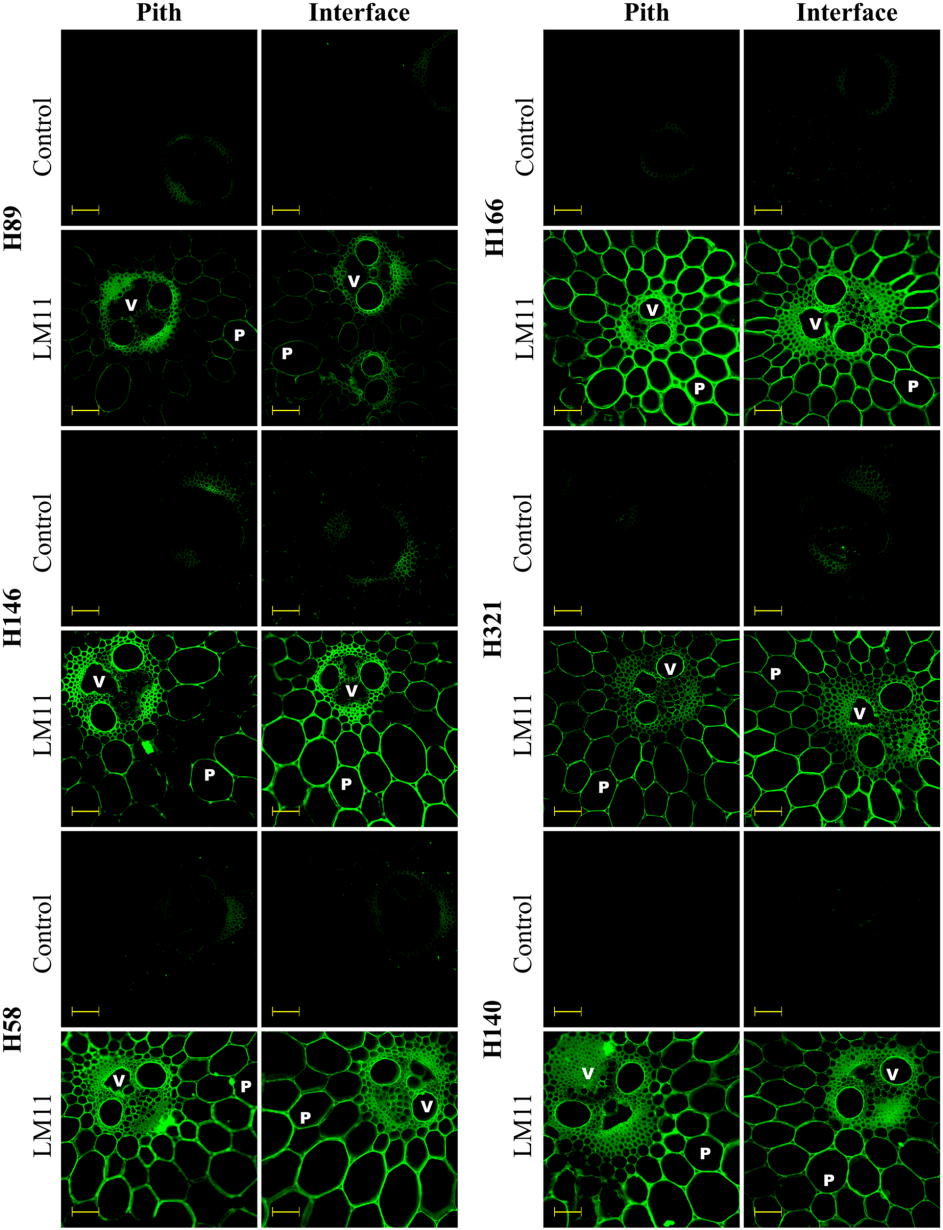
Fluorescence micrographs of pith and pith-rind interface transversal cuts of six different sugarcane hybrids based on indirect immunolabeling analysis for arabinoxylan epitopes labeled with LM11 primary antibody and Alexa Fluor 568 secondary antibody. *V* and *P* indicate vascular bundles and parenchyma, respectively. Control corresponds to the transversal cuts labeled only with the secondary antibody. *Scale bars* correspond to 100 µm

A second antibody that labels arabinoxylans (LM11) [36] was also used to identify xylan distribution along the internodes (Fig. 3). Fluorescence detection showed that LM11 bonded to all cell walls, including parenchyma cells from pith, which contrasted with CRCC-M140 labeling. These data demonstrated that the more heavily arabinosylated xylans present in the pith region are more strongly linked to LM11 than to the CRCC-M140 antibody.

The extensive labeling by LM11 emphasizes that this antibody was also suitable for the general immunological detection of xylans in grasses such as sugarcane. However, the fluorescence levels detected in the rind region were affected by autofluorescence of the highly lignified tissues, even when using appropriate filters to suppress autofluorescence from lignin. Complementarily, CRCC-M140 seems useful to reveal less substituted xylans because it was barely linked to the more decorated xylans present in the parenchyma of the pith region.

**Fig. 4:**
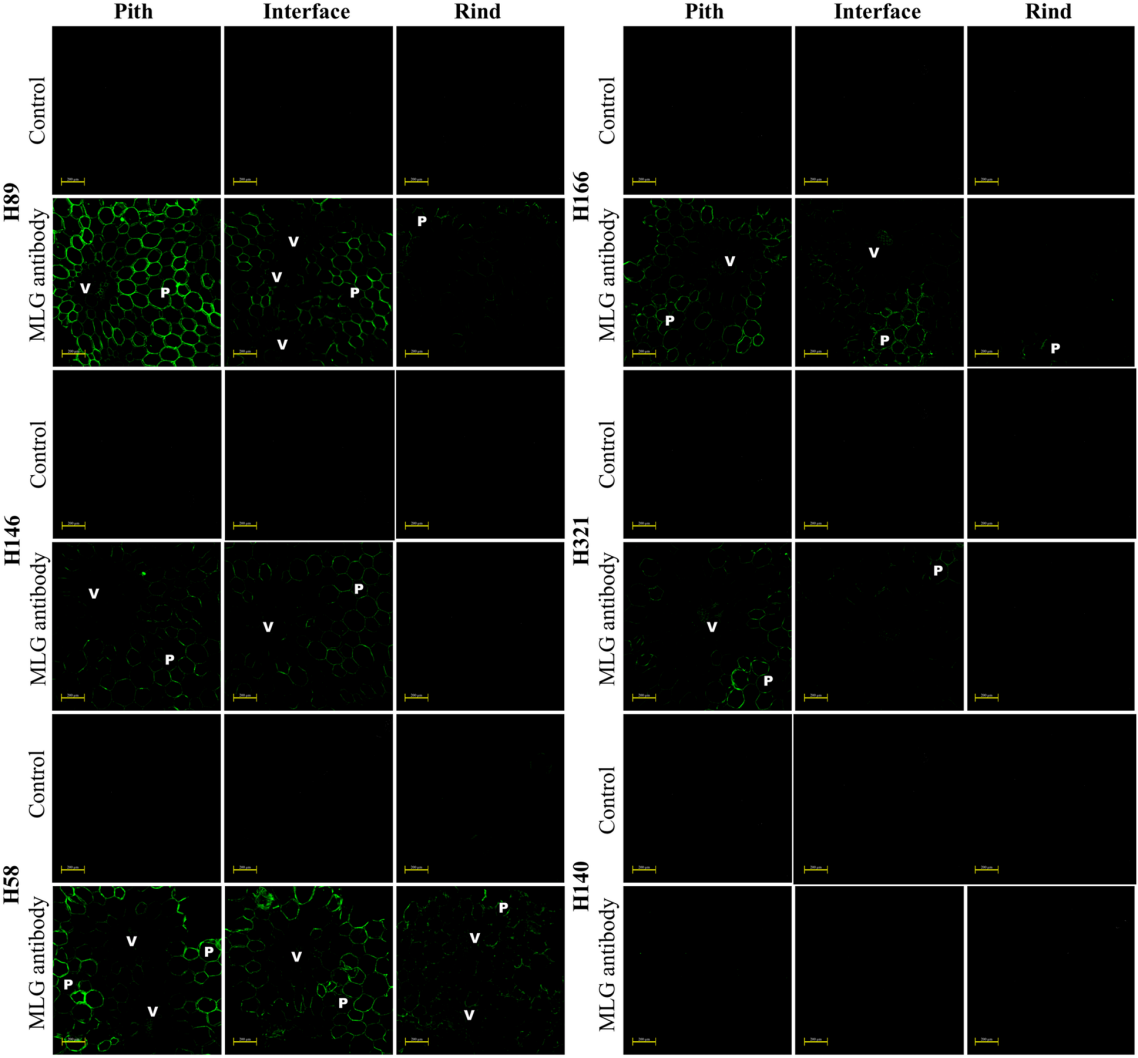
Fluorescence micrographs of pith, pith-rind interface, and rind transversal cuts of six different sugarcane hybrids based on indirect immunolabeling analysis for MLG epitopes labeled with MLG primary antibody and Alexa Fluor 514 secondary antibody. *V* and *P* indicate vascular bundles and parenchyma, respectively. Control corresponds to the transversal cuts labeled only with the secondary antibody. *Scale bars* correspond to 200 µm

The MLG monoclonal antibody mainly labeled parenchyma cell walls from the pith region (Fig. 4). The parenchyma cells from the pith regions of hybrids with low-lignin contents (H89 and H58) showed the strongest labeling, indicating a high accumulation of MLG in these cell walls. Pith-rind interface fractions also showed an evident presence of the MLG epitope in parenchyma cell walls, but very little or no MLG epitopes were detected in parenchyma from rind regions of all hybrids. These results agree with the MLG distribution pattern revealed by immunodot assays (Additional file 2: Figure S1) and the enzyme-based quantification (Fig. 1). Vascular bundles almost did not label (under 50 × magnification) when compared to parenchyma cells because they appeared as dark spots in the fluorescence images (Fig. 4). Similar experiments regarding immunofluorescence in grasses other than sugarcane showed that MLG is mainly deposited in primary cell walls and very little in secondary cell walls [22, 37]. However, some recent reports showed MLG’s presence in fiber cell walls from vascular bundles of developing leaves and mature sclerenchyma tissues from rice stems [23, 38].

**Fig. 5:**
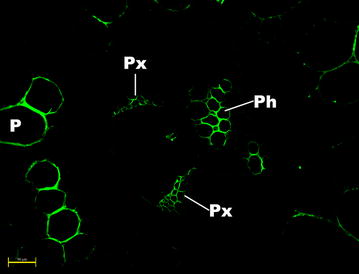
Fluorescence micrographs of a pith transversal cut of the sugarcane hybrid 58 based on indirect immunolabeling analysis for MLG epitopes labeled with MLG primary antibody and Alexa Fluor 514 secondary antibody. *Ph*, *Px*, and *P* indicate phloem, protoxylem, and parenchyma, respectively. *Scale bar* corresponds to 50 µm

**Fig. 6:**
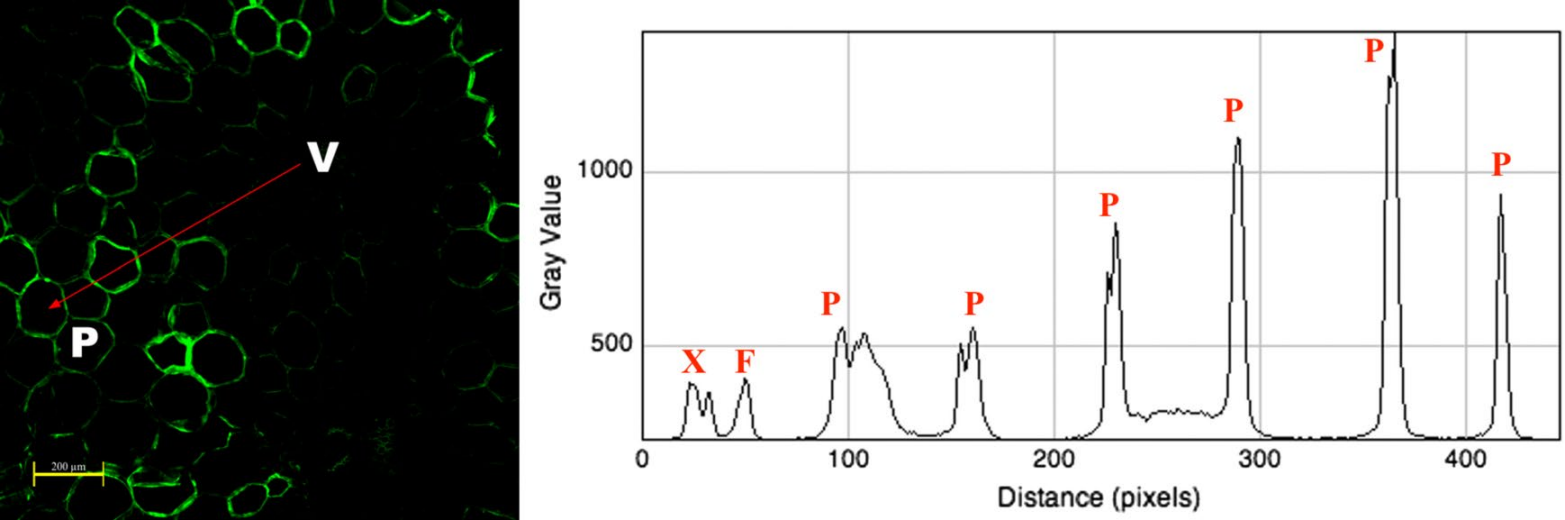
Fluorescence intensity assessment of a pith transversal cut of the sugarcane hybrid 58 based on indirect immunolabeling analysis for MLG epitopes labeled with MLG primary antibody and Alexa Fluor 514 secondary antibody. Fluorescence intensity assessed for cell walls from V to P as indicated by the *red arrow*. X and F correspond to vessels and fibers contained in a vascular bundle xylem, and P corresponds to several parenchyma cell walls. *Scale bar* corresponds to 200 µm

Vascular bundles of sugarcane samples analyzed under a higher magnification (200 ×) showed MLG labeling in specific cells of the phloem and protoxylem (Fig. 5). This differential distribution was only evident in pith regions, indicating that vascular bundles from pith present a particular MLG topochemistry. Interestingly, parenchyma cells right next to the vascular bundles in the pith region presented lower MLG labeling compared to parenchyma cells away from the vascular bundle surroundings (Fig. 6). Leroux et al. [39] found the opposite in fern species. For example, *Asplenium elliottii* stems showed strong MLG antibody binding in parenchyma cells surrounding the vascular bundles and other mechanical tissues. The authors suggested that MLG may have a mechanical function in the cell walls of these species, which might not be the case in sugarcane pith cells.

Comparison of the MLG distribution among several hybrids showed that the main exception to common results was H140. This hybrid showed almost no fluorescence due to MLG in pith and other internode regions. This was corroborated by very low MLG contents detected by the enzyme-based quantification (Fig. 1). Moreover, this hybrid was the most lignified one corroborating that mature-lignified tissues lose MLG. In contrast, the hybrids H58 and H89, which presented the lowest lignin contents (Table 1), provided strong MLG labeling, especially in the pith region. Hybrids with a relatively high amount of xylan (H146, H166, and H321) showed intermediary labeling for MLG.

### Crystalline cellulose in the internode regions

**Fig. 7:**
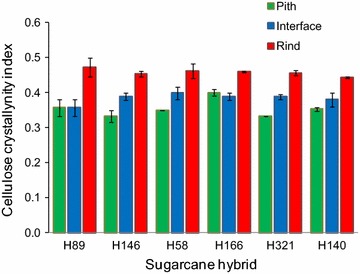
Cellulose crystallinity index estimated by X-ray diffraction of the pith, pith-rind interface, and rind regions of six different sugarcane hybrids. *Error bars* represent the standard deviations for triplicate determinations

The crystallinity index (CI) of each sample was estimated by X-ray diffraction [4]. The CI was calculated using the peak height method, which is useful to provide relative differences between cellulosic samples, such as the different sugarcane internode regions under evaluation (Fig. 7). In every hybrid, the rind region presented the highest estimated CI values (0.44–0.47), whereas the pith regions presented the lowest CI levels (0.33–0.40). The pith-rind interface CI values were intermediate, except in H89 and H166, which showed no difference between the pith and pith-rind interface. The proportion of amorphous cellulose was almost constant in all samples because the height of the minimum between the peaks 101 and 002 at approximately 19 degrees 2θ, assigned to amorphous cellulose [4], did not vary among the samples. High CI values resulted from intense 002 peaks (at 22 degrees 2θ) (see illustrative X-ray diffraction data in Additional file 3: Figure S2).

### Enzymatic hydrolysis of the internode regions

**Fig. 8:**
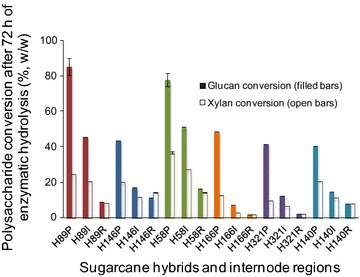
Glucan and xylan enzymatic conversions of the pith (P), pith-rind interface (I), and rind (R) regions of six different sugarcane hybrids after 72 h of hydrolysis with commercial enzymes. *Error bars* represent the standard deviations for triplicate hydrolysis experiments

One of the objectives of the present study was to evaluate whether the hemicellulose distribution in cell walls is correlated with cell wall recalcitrance. Each sugarcane sample was hydrolyzed with a commercial cellulase preparation to evaluate the digestibility of the biomasses without any pretreatment besides milling. The enzymatic glucan conversion pattern showed that the pith fractions were the least recalcitrant in all hybrids (40–85 % of glucan conversion after 72 h hydrolysis), whereas the rind fractions were the most recalcitrant (2–9 % glucan conversion after 72 h hydrolysis) (Fig. 8; Additional file 4: Figure S3, presenting the kinetics of glucan hydrolysis). Pith-rind interfaces showed intermediate recalcitrance (7–46 % glucan conversion after 72 h hydrolysis). This differential distribution of recalcitrance is in accordance with our previous work [26], even though an increased number of sugarcane hybrids was now evaluated. The higher recalcitrance of the rind region of grasses has long been associated with their higher lignin content [7, 9, 26]. Regarding the pith fractions from different sugarcane hybrids, we showed that H58 and H89, which presented low-lignin and hemicellulose contents, also presented the highest glucan conversion levels (78 and 85 % in pith, after 72 h hydrolysis, respectively). In contrast, pith fractions from hybrids with high lignin or hemicellulose contents showed poor glucan conversions (40–49 % in piths, after 72 h hydrolysis).

Xylan hydrolysis followed the same pattern of glucan hydrolysis, with decreasing digestibility toward the rind (Fig. 8; Additional file 5: Figure S4). However, xylan conversion to xylose was lower than the cellulose conversions to glucose for all evaluated samples. To check if the commercial enzymatic cocktail had insufficient xylanase activity, xylanase- and β-xylosidase-amended hydrolyses (10 and 20 IU/g of substrate, respectively) were used, but the xylan to xylose conversion levels were not increased. It is probable that the high degree of xylan substitution (including acetylation) in non-pretreated sugar cane samples limited its digestibility [15]. Xylan conversion to soluble oligosaccharides instead of xylose is probable, but it was not demonstrated in the current work. Comparison of the xylan conversion levels among studied hybrids indicates that H89 and H58 (the lowest in lignin content) showed the highest xylan conversions in pith regions (25 and 37 % after 72 h of hydrolysis, respectively). However, hybrids with high xylan contents that were low in lignin presented low xylan conversion levels (10–20 % in piths from H146, H166 and H321).

**Fig. 9:**
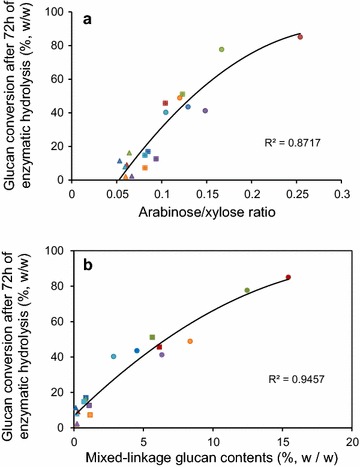
Correlation between glucan conversion after 72 h of enzymatic hydrolysis and the arabinose/xylose ratio in GAX (**a**) and the MLG contents (**b**) of internode fractions from six different sugarcane hybrids. *Different symbol colors* indicate the sugarcane hybrid, as shown in Fig. 8. *Circle*, *square* and *triangle symbols* denote pith, pith-rind interface, and rind, respectively

The current data emphasize that the differential recalcitrance is inherent to several characteristics of the samples. In addition to lignin and xylan contents, xylan substitution level and cellulose crystallinity also affected the cell wall recalcitrance. The xylan substitution with arabinose increased from rind to pith, following the same trend of the samples digestibility (*R*^2^ = 0.87 for a quadratic model—Fig. 9a). The decoration pattern affects the primary hydrolysis of hemicelluloses by enzymes. Branched polymers are more resistant than less decorated polymers [2, 15]. However, GAX with fewer side groups allows for stronger interactions between GAX and cellulose [16]. In the sugar cane hybrids, high arabinose/xylose ratios observed in pith regions of the low-lignin content hybrids (H89 and H58) seemed to decrease cellulose–hemicellulose interaction, contributing to decrease the recalcitrance of these samples. MLG appeared to be one additional factor contributing to the inherent differential distribution of recalcitrance among internode regions and sugarcane hybrids. The MLG content plotted against digestibility of each sample after 72 h hydrolysis resulted in a high data fitting (*R*^2^ = 0.94 for a quadratic model—Fig. 9b), corroborating that the recalcitrance of sugarcane internode regions relies not only on lignin and xylan contents but also on hemicellulose composition, especially when the glucan fraction had a significant contribution from MLG. This unique polysaccharide could therefore be used as a titer for recalcitrance in sugarcane materials, as low recalcitrant regions, such as pith from H58 and H89, were fairly rich in MLG. Moreover, we investigated whether MLG could contribute as a source of glucose monomers when treated by commercial cellulases. The results indicated that 88 % of the MLG was converted into glucose within only 4 h of enzymatic digestion (Additional file 6: Figure S5). Vega-Sánchez et al. [18], for instance, engineered *Arabidopsis thaliana* plants to promote MLG accumulation in cell walls under the control of a senescence-associated promoter. The biomass obtained from the engineered plants showed up to 42 % increased digestibility compared to control plants.

In a previous study, we developed a calculated parameter based on glucan availability and the vascular bundle distribution to predict recalcitrance in non-pretreated sugarcane samples [26]. With the currently increased dataset, including six sugarcane hybrids in a total of 18 samples and a detailed mapping of hemicelluloses, it become clear that the high recalcitrance observed in the sugarcane internode regions rich in vascular bundles (rind) can be assigned to several complimentary characteristics, including the well-documented embedding of cellulose microfibrils by high contents of lignin and xylan. Additionally, we observed that low MLG contents, xylan with less substituted structures and high proportions of crystalline cellulose also contributed to the recalcitrance of rind regions. In contrast, less recalcitrant tissues found in the pith region were less lignified, contained a higher proportion of MLG, presented lower cellulose CI values and xylans with a higher degree of substitution. All of these factors seemed to contribute to a cell wall with less organized and less embedded cellulose, which was more susceptible to enzymatic hydrolysis.

## Conclusions

GAX and MLG were the first and second most abundant hemicelluloses in a variety of sugarcane hybrids. Xylans were predominant in all cell walls, despite the fact that highly substituted GAX and MLG were relevant in the parenchyma cell walls of the innermost regions of the sugarcane internodes. MLG particularly accumulated in the pith region of the low-lignin content hybrids. The compiled data indicate that high recalcitrance can be assigned to the collective characteristics of the internode regions from the varied sugarcane hybrids. Cellulose-embedding components, such as lignin and xylan, are critical for increased recalcitrance, while MLG and highly substituted GAX contribute to the generation of a less recalcitrant material. From the studied hybrids, H89 and H58 join biomass characteristics useful for low recalcitrance (low-lignin and high MLG contents, and more substituted GAX). These characteristics can be used by breeders to select subsequent generation of plants aiming low recalcitrance in the lignocellulosic biomass.

## Methods

### Raw material and biomass preparation

Six sugarcane hybrids with varied lignin and hemicellulose contents were selected from a larger set of plants [40]. The vegetative propagation of each hybrid was performed as described previously [26]. The plants used in the current work originated from the second regrow of original plants cut in July 2012. From July 2012, each planted row received 100 g of 4:14:8 NPK (nitrogen, phosphorous, and potassium) fertilizer with subsequent fertilizations performed with 100 g of 10:10:10 NPK every 3 months until harvesting. Harvesting occurred in June 2013 corresponding to 12-month-old mature sugarcane stems. Internodes 3–7 (from the plant base) were separated from every stem and stored at −18 °C. These internodes were cut into 30-mm circular pieces following the longitudinal axis of the stem. Then, the 30-mm pieces were divided into fractions from the periphery to the center as follows: (a) the first external 2 mm containing epidermis, cortical cells, and part of the outermost rind (outermost fraction); (b) the remaining material was measured and divided into three equal segments, which were rind, pith–rind interface and pith fractions (from exterior to interior). Only pith, pith-rind interface, and rind were used in the present work. All samples were extracted with water in a Soxhlet apparatus in 8-h cycles to remove sucrose. After each extraction cycle, total sugars in the extract were measured by a phenol–sulfuric acid assay [41]. Extraction cycles were performed with distilled water until the extract was free of soluble sugars (on average, five cycles).

For microscopic studies, five pieces of each fraction were randomly selected from each hybrid and cut into small blocks (approximately 10 × 5 × 5 mm), which were stored at 4 °C in water. The remaining of the extracted samples were air-dried and stored in dry conditions until use. Air-dried samples were used for enzymatic digestion, chemical composition assays, immunodot analyses, enzyme-based MLG determination, and crystallinity index evaluation.

### Chemical characterization of the samples

Air-dried samples were milled to pass through a 0.84-mm screen and used for the determination of chemical composition. Approximately, 1 g of the milled sample was extracted with 95 % ethanol for 6 h in a Soxhlet apparatus. The percentage of ethanol-soluble extractives was determined on the basis of the dry weights of the extracted and non-extracted milled samples. This procedure was performed in triplicate and the average values and standard deviations are reported in the text.

For quantitative determination of polysaccharides and lignin, ethanol-extracted samples were hydrolyzed with sulfuric acid following procedures previously described [42]. This experiment was conducted in triplicate. Average values followed by standard deviations are reported in the text. The factors used to convert sugar monomers to anhydromonomers were 0.90 for glucose and 0.88 for xylose and arabinose. The acetyl content was calculated as the acetic acid content multiplied by 0.72.

For evaluation of the non-cellulosic polysaccharides, the 0.84-mm milled samples were further milled in a steel ball mill operating at 30 s^−1^ frequency for 2.5–4 min. Milled samples were previously treated to remove starch as described elsewhere [43]. Treated material was digested with 2 mol/L trifluoroacetic acid (TFA) at 121 °C for 1 h. Released sugars were assayed by HPAEC according to the methods described in Øbro et al. [28].

### MLG detection by immunodot assay and enzyme-based quantification

For immunodot assays, ball-milled samples were treated at 10 mg/mL with 4 M KOH for 24 h under 600 rpm at room temperature to isolate crude hemicellulose extracts. The reaction tubes were then centrifuged and the supernatant containing the hemicelluloses was transferred to another tube. This fraction was neutralized with 8.7 mol/L acetic acid, followed by serial 1:10 dilutions in 0.8 mol/L KOH. The diluted fractions were spotted on nitrocellulose membranes (0.2 µm Sigma-Aldrich, St. Louis, MO, EUA) with two replicates. Solutions were allowed to absorb on the membrane for 5 min, washed with phosphate-buffered saline (PBS, pH 7.4) for 5 min and blocked with PBS containing 5 % non-fat milk for 1 h (MP/PBS). The membranes were then incubated overnight at 4 °C with primary monoclonal antibody for MLG diluted 1:10,000 in MP/PBS [44]. Afterward, the membrane was washed three times with PBS for 5 min and then incubated for 1 h with a secondary antibody (Goat-polyclonal antibody to mouse—Horseradish Peroxidase—Sigma-Aldrich, St. Louis, MO, EUA) diluted 1:15,000 in MP/PBS. After incubation, the membrane was washed four times with PBS for 5 min and developed with the detection substrate (Supersignal West Dura Extended Duration Substrate—Thermo Scientific, Waltham, MA, EUA). Barley MLG (Megazyme, Ireland) was used as a positive control.

For quantitative MLG determination, ball-milled samples were assayed using a MLG-assay kit and procedures (Megazyme, Ireland). The procedure involved the extraction of MLG from ball-milled samples with water at 100 °C for 3 min. The supernatants were digested with lichenase, which releases short glucan oligosaccharides that are then hydrolyzed to glucose by ß-glucosidase. Glucose was quantified by the glucose oxidase/peroxidase assay.

### Immunofluorescence microscopy

For immunofluorescence microscopy, each sample was sectioned using a Leica vibratome (VT1000S, Leica, Wetzlar, Germany) to yield 60–80-µm transversal cuts. The sections were washed three times in PBS using a 24-well plate and blocked with MP/PBS for 1 h. The sections were then incubated with a primary monoclonal antibody for 1 h. The samples were probed with three different monoclonal antibodies: (a) xylan CRCC M140 antibody [35] diluted 1:10 in MP/PBS; (b) arabinoxylan LM11 antibody [36] diluted 1:10 in MP/PBS; and (c) MLG antibody [44] diluted 1:1500 in MP/PBS. After antibody binding, the samples were washed three times with PBS and incubated with secondary antibodies (Alexa Fluor 514—Sigma/Aldrich anti-mouse for CRCC M140 and MLG and Alexa Fluor 568—Sigma/Aldrich anti-rat for LM11), both diluted 1:10,000 in MP/PBS. The sections were visualized with a Leica microscope (DM 4000 B) under YFP filter for Alexa Fluor 514 and TX2 filter for Alexa Fluor 568. Sections treated only with secondary antibody were used as negative controls. Images were processed with ImageJ software (version 10.2).

### Determination of crystallinity index

The air-dried milled samples were analyzed in an X-ray diffractometer (Empyrean copper-radiation model, Panalytical, The Netherlands). The measurement was carried out according to the peak height method [4], as follows:

$${\text{CI}} = ({\text{I}}_{002}-{\text{I}}_{\text{am}})/{\text{I}}_{002},$$

where CI is the crystallinity index, I_002_ is the intensity of the peak 002 at approximately 22 degrees 2θ, and I_am_ is the intensity measured at the minimum between the peaks 101 and 002 at approximately 19 degrees 2θ assigned to amorphous cellulose.

### Enzymatic hydrolysis

Enzymatic hydrolysis assays were performed using commercial enzyme preparation (Cellic Ctec2 from Novozymes A/S, Denmark) at the dosage of 35 mg protein/g of substrate in all experiments. The hydrolysis reaction was performed in 2 mL screw-capped tubes containing 20 mg of air-dried and milled substrate and the enzyme mixture in a final volume of 1 mL of 100 mM citrate buffer, pH 5.0, containing 0.01 % (w/v) sodium azide. The tubes were incubated at 45 ± 2 °C under 120 rpm. The reaction was stopped at defined periods from 4 to 72 h by cooling down the reaction in an ice bath. The tube content was centrifuged at 3400*g* for 15 min at 10 °C. Twenty microliters of the supernatant was sampled and diluted with distilled water to assay glucose and xylose by HPLC (Waters Corporation, Milford, MA, USA) using an HPX87H column (Bio-Rad, Hercules, CA, USA) at 45 °C eluted with 5 mmol/L sulfuric acid at 0.6 mL/min. Sugars were detected with a refractive index detector (model 2414, Waters Corporation, Millford, MA, USA) set at 35 °C. A control sample from each experiment was obtained just after enzyme addition (reaction time zero). Glucose and xylose detected in this sample were subtracted from the sugar contents released by enzymatic hydrolysis. Barley MLG (Megazyme, Ireland) was treated with Cellic Ctec2 under identical conditions, replacing the biomass by MLG. To check if the commercial enzymatic cocktail had insufficient xylanase activity, GH-10 xylanase from *Cellvibrio mixtus* (Megazyme, Ireland) and GH-43 β-xylosidase from *Selenomonas ruminantium* (Megazyme, Ireland) were added to conventional hydrolysis experiments at 10 and 20 IU/g of substrate, respectively. Triplicates were used for all hydrolysis experiments.

## Additional files

10.1186/s13068-016-0513-2 Monomeric sugars released from internode fractions from six different sugarcane hybrids after mild hydrolysis with 2 mol/L trifluoroacetic acid (TFA). TFA hydrolysis barely hydrolyzes crystalline cellulose allowing for the evaluation of non-cellulosic monosaccharides. Xylose, followed by glucose and arabinose was the most abundant monosaccharides.
10.1186/s13068-016-0513-2 Immunodot analysis with a MLG monoclonal antibody of 4 mol/L KOH extracts prepared from internode fractions of six different sugarcane hybrids. For each sample, three different extract dilutions were prepared and assessed in duplicate. Dark spots indicate a positive reaction with the MLG monoclonal antibody.
10.1186/s13068-016-0513-2 Illustrative X-ray diffraction spectra of the rind (red) and pith (green) regions of the sugarcane hybrid 58. The minimum between the peaks at approximately 19 degrees 2θ is assigned to amorphous cellulose, whereas the intense peaks at 22 degrees 2θ is assigned to one of the crystalline forms of cellulose.
10.1186/s13068-016-0513-2 Time dependence of the enzymatic conversion of glucan to glucose in internode fractions from six different sugarcane hybrids.
10.1186/s13068-016-0513-2 Time dependence of the enzymatic conversion of xylan to xylose in internode fractions from six different sugarcane hybrids.
10.1186/s13068-016-0513-2 Time dependence of the enzymatic conversion of barley MLG to glucose monomers with commercial enzymes.

## Abbreviations

GAX: acetylated glucuronoarabinoxylan
MLG: mixed-linkage glucan
TFA: trifluoroacetic acid
CI: crystallinity index
HPAEC: high-performance anion-exchange chromatography
PBS: phosphate-buffered saline

## References

[1] Himmel ME, Ding S-H, Johnson DK, Adney WS, Nimlos MR, Brady JW, Foust TD (2007). Biomass recalcitrance: engineering plants and enzymes for biofuels production. Science..

[2] DeMartini JD, Pattathil S, Miller JS, Li H, Hahn MG, Wyman CE (2013). Investigating plant cell wall components that affect biomass recalcitrance in poplar and switchgrass. Energy Environ Sci..

[3] McCann MC, Carpita NC (2015). Biomass recalcitrance: a multi-scale, multi-factor, and conversion-specific property. J Exp Bot..

[4] Park S, Baker JO, Himmel ME, Parilla PA, Johnson DK (2010). Cellulose crystallinity index: measurement techniques and their impact on interpreting cellulase performance. Biotechnol Biofuels..

[5] Arantes V, Saddler JN (2010). Access to cellulose limits the efficiency of enzymatic hydrolysis: the role of amorphogenesis. Biotechnol Biofuels..

[6] Hu JG, Arantes V, Pribowo A, Gourlay K, Saddler JN (2014). Substrate factors that influence the synergistic interaction of AA9 and cellulases during the enzymatic hydrolysis of biomass. Energy Environ Sci..

[7] Jung HG, Casler MD (2006). Maize stem tissues: impact of development on cell wall degradability. Crop Sci..

[8] Siqueira G, Milagres AMF, Carvalho W, Koch G, Ferraz A (2011). Topochemical distribution of lignin and hydroxycinnamic acids in sugar-cane cell walls and its correlation with the enzymatic hydrolysis of polysaccharides. Biotechnol Biofuels.

[9] Zheng MJ, Ximenes E, Ladisch MR, Mosier NS, Vermerris W, Huang CP, Sherman DM (2012). Tissue-specific biomass recalcitrance in corn stover pretreated with liquid hot-water: enzymatic hydrolysis (part 1). Biotechnol Bioeng..

[10] Ding S-Y, Liu Y-S, Zeng Y, Himmel ME, Baker JO, Bayer EA (2012). How does plant cell wall nanoscale architecture correlate with enzymatic digestibility?. Science..

[11] Ferraz A, Costa THF, Siqueira G, Milagres AMF, Silva SS, Chandel AK (2014). Mapping of cell wall components in lignified biomass as a tool to understand recalcitrance. Biofuels in Brazil: fundamental aspects, recent developments, and future perspectives.

[12] Mansfield SD, Mooney C, Saddler JN (1999). Substrate and enzyme characteristics that limit cellulose hydrolysis. Biotechnol Prog..

[13] Zhao X, Zhang L, Liu D (2012). Biomass recalcitrance. Part I: The chemical compositions and physical structures affecting the enzymatic hydrolysis of lignocellulose. Biofuel Bioprod Bior.

[14] Pu YQ, Hu F, Huang F, Davison BH, Ragauskas AJ (2013). Assessing the molecular structure basis for biomass recalcitrance during dilute acid and hydrothermal pretreatments. Biotechnol Biofuels..

[15] Várnai A, Costa THF, Faulds CB, Milagres AMF, Siika-aho M, Ferraz A (2014). Effects of enzymatic removal of plant cell wall acylation (acetylation, *p*-coumaroylation, and feruloylation) on accessibility of cellulose and xylan in natural (non-pretreated) sugar cane fractions. Biotechnol Biofuels..

[16] Vogel J (2008). Unique aspects of the grass cell wall. Curr Opin Plant Biol..

[17] Burton RA, Fincher GB (2009). (1,3;1,4)-ß-D-glucans in cell walls of the Poaceae, lower plants, and fungi: a tale of two linkages. Mol Plant..

[18] Vega-Sánchez ME, Loqué D, Lao J, Catena M, Verhertbruggen Y, Herter T, Yang F, Harholt J, Ebert B, Baidoo EEK, Keasling JD, Scheller HV, Heazlewood JL, Ronald PC (2015). Engineering temporal accumulation of a low recalcitrance polysaccharide leads to increased C6 sugar content in plant cell walls. Plant Biotechnol J..

[19] Kiemle SN, Zhang X, Esker AR, Toriz G, Gatenholm P, Cosgrove DJ (2014). Role of (1,3)(1,4)-β-glucan in cell walls: interaction with cellulose. Biomacromolecules..

[20] Carpita NC, Defernez M, Findlay K, Wells B, Shoue DA, Catchpole G, Wilson RH, McCann MC (2001). Cell wall architecture of the elongating maize coleoptile. Plant Physiol..

[21] Kozlova LV, Snegireva AV, Gorshkova TA (2012). Distribution and structure of mixed linkage glucan at different stages of elongation of maize root cells. Russ J Plant Physiol..

[22] Trethewey JAK, Campbell LM, Harris PJ (2005). (1–3), (1–4)-ß-D-glucans in the cell walls of the poales (sensu lato): an immunogold labeling study using a monoclonal antibody. Am J Bot..

[23] Vega-Sánchez ME, Verhertbruggen Y, Scheller HV, Ronald PC (2013). Abundance of mixed linkage glucan in mature tissues and secondary cell walls of grasses. Plant Signal Behav..

[24] Grabber JH, Panciera MT, Hatfield RD (2002). Chemical composition and enzymatic degradability of xylem and nonxylem walls isolated from alfalfa internodes. J Agr Food Chem..

[25] Barros-Rios J, Santiago R, Malvara RA (2012). Jung H-JG. Chemical composition and cell wall polysaccharide degradability of pith and rind tissues from mature maize internodes. Anim Feed Sci Technol..

[26] Costa THF, Masarin F, Bonifácio TO, Milagres AMF, Ferraz A (2013). The enzymatic recalcitrance of internodes of sugar cane hybrids with contrasting lignin contents. Ind Crop Prod..

[27] Mendes FM, Fonseca MB, Ferraz A, Milagres AMF (2016). Anatomic and ultrastructural characteristics of different regions of sugar cane internodes affect their response to alkaline-sulfite pretreatment and material recalcitrance. Energy Fuels..

[28] ØBro J, Harholt J, Scheller HV, Orfila C (2004). Rhamnogalacturonan I in *Solanum tuberosum* tubers contains complex arabinogalactan structures. Phytochemistry.

[29] Harholt J, Suttangkakul A, Scheller HV (2010). Biosynthesis of Pectin. Plant Physiol..

[30] Scheller HV, Ulvskov P (2010). Hemicelluloses. Annu Rev Plant Biol..

[31] Carpita NC (1984). Cell wall development in maize coleoptiles. Plant Physiol..

[32] Obel N, Porchia AC, Scheller HV (2002). Dynamic changes in cell wall polysaccharides during wheat seedling development. Phytochemistry..

[33] Gibeaut DM, Pauly M, Bacic A, Fincher GB (2005). Changes in cell wall polysaccharides in developing barley (*Hordeum vulgare*) coleoptiles. Planta..

[34] Christensen U, Alonso-Simon A, Scheller HV, Willats WGT, Harholt J (2010). Characterization of the primary cell walls of seedlings of *Brachypodium distachyon*—a potential model plant for temperate grasses. Phytochemistry..

[35] Pattathil S, Avci U, Baldwin D, Swennes AG, McGill JA, Popper Z, Bootten T, Albert A, Davis RH, Chennareddy C, Dong R, O’Shea B, Rossi R, Leoff C, Freshour G, Narra R, O’Neil M, York WS, Hahn MG (2010). A comprehensive toolkit of plant cell wall glycan-directed monoclonal antibodies. Plant Physiol..

[36] McCartney L, Marcus SE, Knox JP (2005). Monoclonal antibodies to plant cell wall xylans and arabinoxylans. J Histochem Cytochem..

[37] Trethewey JAK, Harris PJ (2002). Location of (1–3)- and (1–3), (1–4)-ß-d-glucans in vegetative cell walls of barley (*Hordeum vulgare*) using immunolabelling. New Phytol..

[38] Vega-Sánchez ME, Verhertbruggen Y, Christensen U, Chen X, Sharma V, Varanasi P, Jobling SA, Talbot M, White RG, Joo M, Singh S, Auer M, Scheller HV, Ronald PC (2012). Loss of *Cellulose synthase-like F6* function affects mixed-linkage glucan deposition, cell wall mechanical properties, and defense responses in vegetative tissues of rice. Plant Physiol..

[39] Leroux O, Sorensen I, Marcus SE, Viane RLL, Willats WGT, Knox JP (2015). Antibody-based screening of cell wall matrix glycans in ferns reveals taxon, tissue and cell-type specific distribution patterns. BMC Plant Biol..

[40] Masarin F, Gurpilhares DB, Baffa DCF, Barbosa MHP, Carvalho W, Ferraz A, Milagres AMF (2011). Chemical composition and enzymatic digestibility of sugarcane clones selected for varied lignin content. Biotechnol Biofuels..

[41] Dubois M, Gilles KA, Hamilton JK, Rebers P, Smith F (1956). Colorimetric method for determination of sugars and related substances. Anal Chem..

[42] Ferraz A, Baeza J, Rodriguez J, Freer J (2000). Estimating the chemical composition of biodegraded pine and eucalyptus wood by DRIFT spectroscopy and multivariate analysis. Bioresource Technol..

[43] Harholt J, Jensen JK, Sørensen SO, Orfila C, Pauly M, Scheller HV (2006). Arabinan deficient 1 is a putative arabinosyltransferase involved in biosynthesis of pectic arabinan in Arabidopsis. Plant Physiol..

[44] Meikle PJ, Hoogenraad NJ, Bonig I, Clarke AE, Stone BA (1994). A (1-3,1-4)-beta-glucan-specific monoclonal antibody and its use in the quantitation and immunocytochemical location of (1-3,1-4)-beta-glucans. Plant J..

