# Supplementary material for: Tissue-specific distribution of hemicelluloses in six different sugarcane hybrids as related to cell wall recalcitrance
Source: Biotechnol Biofuels. 2016 May 4;9:99. doi: 10.1186/s13068-016-0513-2 (PMC4855430; doi:10.1186/s13068-016-0513-2)
Supplement: Supplementary file 1 — 10.1186/s13068-016-0513-2 Monomeric sugars released from internode fractions from six different sugarcane hybrids after mild hydrolysis with 2 mol/L trifluoroacetic acid (TFA). TFA hydrolysis barely hydrolyzes crystalline cellulose allowing for the evaluation of non-cellulosic monosaccharides. Xylose, followed by glucose and arabinose was the most abundant monosaccharides. [file 13068_2016_513_MOESM1_ESM.pdf]

Table S1. Monomeric sugars released from internode fractions from six different sugarcane hybrids after mild hydrolysis with 2 mol.L^{-1} trifluoracetic acid (TFA)

Sugarcane hybrids	Internode region	Monomeric sugars released by mild TFA hydrolysis (%, w/w on oven dry basis)						
		Rhamnose	Arabinose	Galactose	Glucose	Xylose	Galacturonic acid	Glucuronic acid
H 89	Pith	0.15 ± 0.02	3.2 ± 0.6	0.8 ± 0.2	12 ± 2	11 ± 2	0.5 ± 0.1	trace
	Interface	0.14 ± 0.01	2.7 ± 0.1	0.6 ± 0.1	8.8 ± 0.8	12.6 ± 0.5	0.4 ± 0.1	trace
	Rind	0.09 ± 0.01	1.9 ± 0.1	0.3 ± 0.1	4.3 ± 0.4	15 ± 1	0.4 ± 0.1	nd
H 146	Pith	0.17 ± 0.04	3.7 ± 0.7	0.9 ± 0.2	12.3 ± 0.2	9 ± 1	0.7 ± 0.1	trace
	Interface	0.11 ± 0.01	2.6 ± 0.2	0.6 ± 0.1	8.7 ± 0.7	13 ± 1	0.5 ± 0.1	trace
	Rind	nd	1.9 ± 0.2	0.3 ± 0.1	2.7 ± 0.9	14 ± 1	0.3 ± 0.1	nd
H 58	Pith	0.13 ± 0.02	2.9 ± 0.1	0.7 ± 0.1	6.3 ± 0.2	15.2 ± 0.9	0.5 ± 0.1	trace
	Interface	0.09 ± 0.04	2.3 ± 0.7	0.4 ± 0.1	1.9 ± 0.5	16 ± 4	0.5 ± 0.1	nd
	Rind	0.09 ± 0.01	2.1 ± 0.1	0.3 ± 0.1	0.9 ± 0.2	17.4 ± 0.7	0.4 ± 0.1	nd
H 166	Pith	0.16 ± 0.03	3.4 ± 0.7	0.7 ± 0.2	8 ± 1	15 ± 2	0.6 ± 0.1	trace
	Interface	0.09 ± 0.01	2.4 ± 0.2	0.4 ± 0.1	4.1 ± 0.6	16 ± 1	nd	nd
	Rind	nd	1.7 ± 0.1	0.2 ± 0.1	2.2 ± 0.4	17.2 ± 0.9	0.4 ± 0.1	nd
H 321	Pith	0.12 ± 0.01	2.9 ± 0.1	0.7 ± 0.1	11.5 ± 0.8	13.1 ± 0.6	0.5 ± 0.1	trace
	Interface	0.09 ± 0.01	2.5 ± 0.1	0.4 ± 0.1	4.8 ± 0.3	16.0 ± 0.9	0.4 ± 0.1	nd
	Rind	0.07 ± 0.02	1.8 ± 0.3	0.3 ± 0.1	1.6 ± 0.3	15 ± 2	0.2 ± 0.1	nd
H 140	Pith	0.15 ± 0.01	3.9 ± 0.1	1.1 ± 0.1	9.2 ± 0.3	16.2 ± 0.3	0.5 ± 0.1	nd
	Interface	0.10 ± 0.01	2.8 ± 0.3	0.7 ± 0.1	4.4 ± 0.9	16.5 ± 0.9	0.4 ± 0.1	nd
	Rind	0.13 ± 0.01	3.0 ± 0.1	0.7 ± 0.1	4.5 ± 0.5	17.9 ± 0.5	0.4 ± 0.1	nd

nd = not detcted; fucose was not detected in the evaluated samples
