# Supplementary material for: Tissue-specific distribution of hemicelluloses in six different sugarcane hybrids as related to cell wall recalcitrance
Source: Biotechnol Biofuels. 2016 May 4;9:99. doi: 10.1186/s13068-016-0513-2 (PMC4855430; doi:10.1186/s13068-016-0513-2)
Supplement: Supplementary file 2 — 10.1186/s13068-016-0513-2 Immunodot analysis with a MLG monoclonal antibody of 4 mol/L KOH extracts prepared from internode fractions of six different sugarcane hybrids. For each sample, three different extract dilutions were prepared and assessed in duplicate. Dark spots indicate a positive reaction with the MLG monoclonal antibody. [file 13068_2016_513_MOESM2_ESM.pdf]

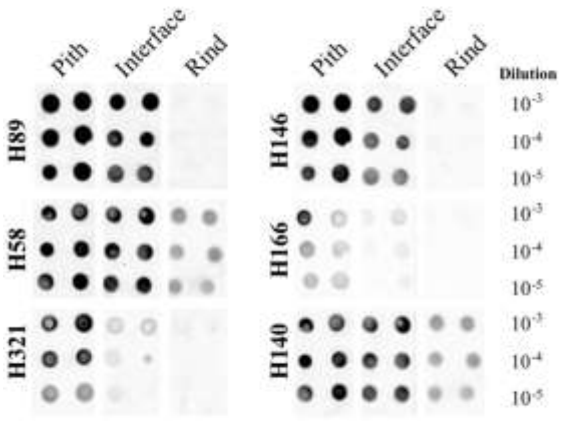

Fig. S1. Immunodot analysis with a MLG monoclonal antibody of 4 mol.l^{-1}-KOH extracts prepared from internode fractions of six different sugarcane hybrids. For each sample, three different extract dilutions were prepared and assessed in duplicate.
