# Supplementary material for: Tissue-specific distribution of hemicelluloses in six different sugarcane hybrids as related to cell wall recalcitrance
Source: Biotechnol Biofuels. 2016 May 4;9:99. doi: 10.1186/s13068-016-0513-2 (PMC4855430; doi:10.1186/s13068-016-0513-2)
Supplement: Supplementary file 3 — 10.1186/s13068-016-0513-2 Illustrative X-ray diffraction spectra of the rind (red) and pith (green) regions of the sugarcane hybrid 58. The minimum between the peaks at approximately 19 degrees 2θ is assigned to amorphous cellulose, whereas the intense peaks at 22 degrees 2θ is assigned to one of the crystalline forms of cellulose. [file 13068_2016_513_MOESM3_ESM.pdf]

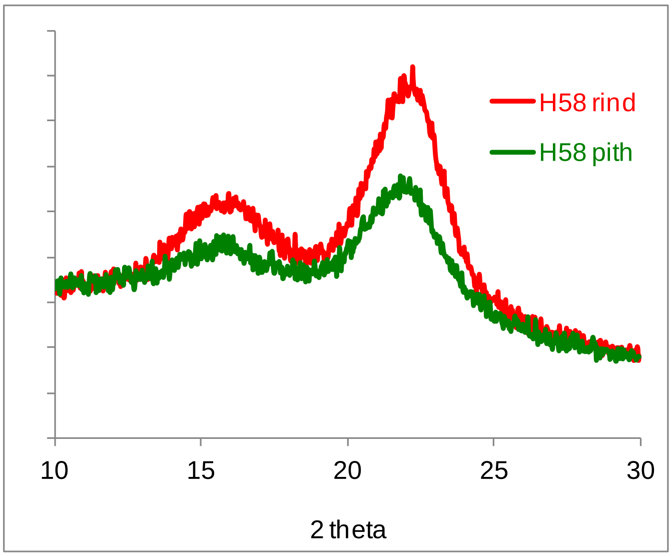


**Fig. S2.** Illustrative X-ray diffraction spectra of the rind (red) and pith (green) regions of the sugarcane hybrid 58.
