# Supplementary material for: Tissue-specific distribution of hemicelluloses in six different sugarcane hybrids as related to cell wall recalcitrance
Source: Biotechnol Biofuels. 2016 May 4;9:99. doi: 10.1186/s13068-016-0513-2 (PMC4855430; doi:10.1186/s13068-016-0513-2)
Supplement: Supplementary file 5 — 10.1186/s13068-016-0513-2 Time dependence of the enzymatic conversion of xylan to xylose in internode fractions from six different sugarcane hybrids. [file 13068_2016_513_MOESM5_ESM.pdf]

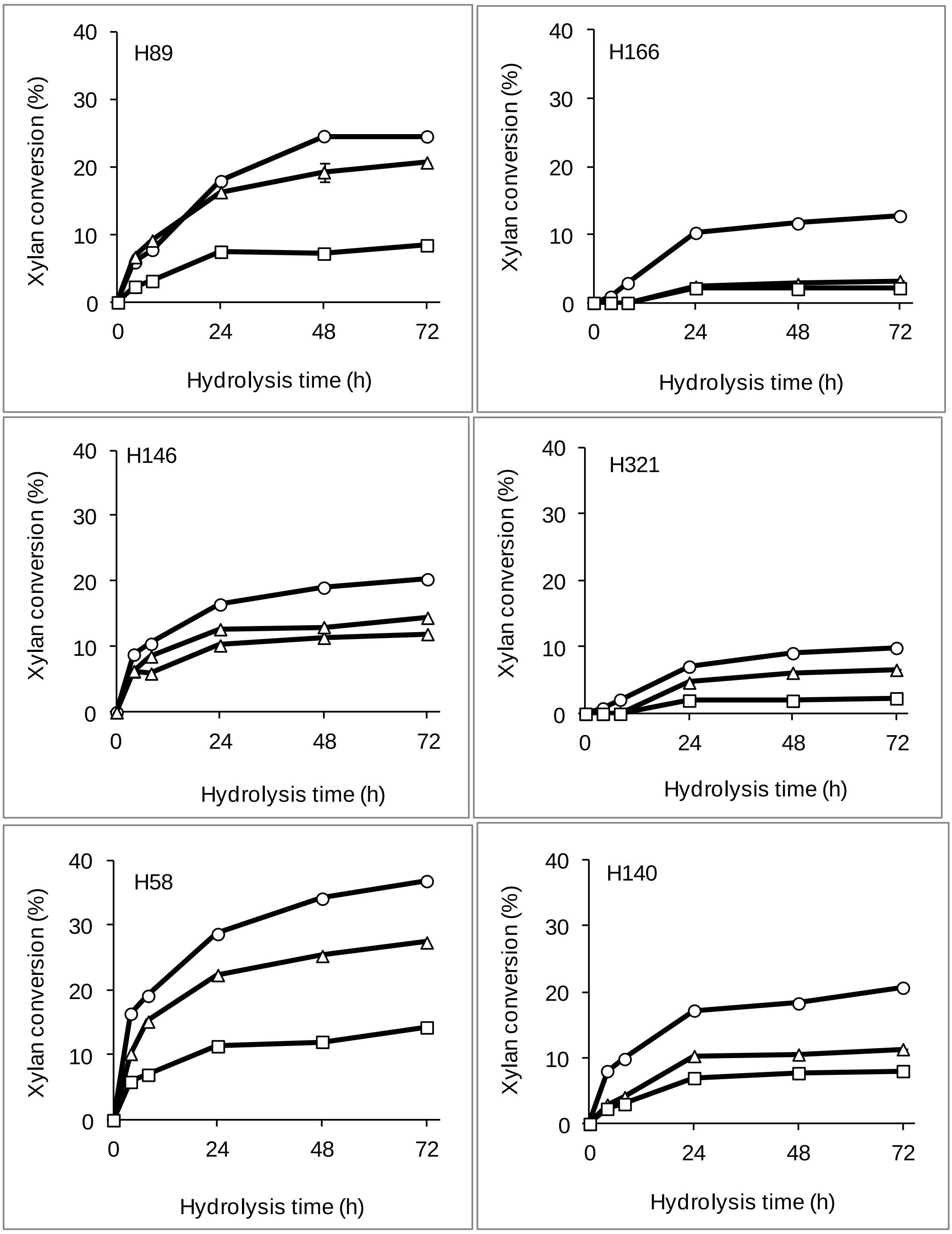

Fig. S4. Time-dependence of the enzymatic conversion of xylan to xylose in internode fractions from six different sugarcane hybrids. Error bars represent the standard deviations for triplicate hydrolysis experiments. Symbols: (circles) pith, (triangles) pith-rind interface, (squares) rind.
