# Supplementary material for: Tissue-specific distribution of hemicelluloses in six different sugarcane hybrids as related to cell wall recalcitrance
Source: Biotechnol Biofuels. 2016 May 4;9:99. doi: 10.1186/s13068-016-0513-2 (PMC4855430; doi:10.1186/s13068-016-0513-2)
Supplement: Supplementary file 6 — 10.1186/s13068-016-0513-2 Time dependence of the enzymatic conversion of barley MLG to glucose monomers with commercial enzymes. [file 13068_2016_513_MOESM6_ESM.pdf]

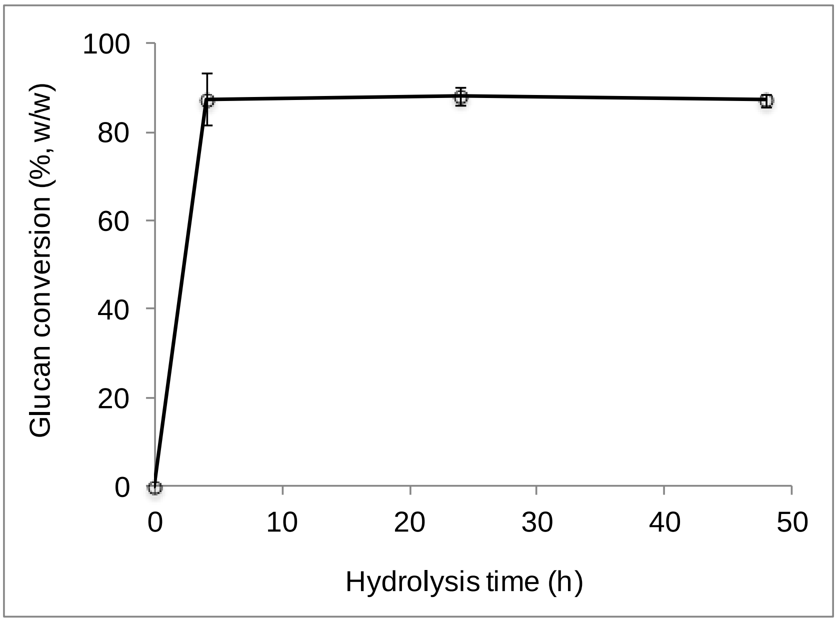

Fig. S5. Time dependence of the enzymatic conversion of barley MLG to glucose monomers with commercial enzymes. Error bars represent the standard deviations for triplicate hydrolysis experiments.
